# Clinical evaluation of two techniques of soft tissue graft removal (free gingival graft/de-epithelialized and linear/subepithelial technique) from the palate: A prospective cohort study

**DOI:** 10.34172/japid.025.3548

**Published:** 2025-09-13

**Authors:** Eduardo Moreira Lessa, Gustavo Vicentis Oliveira Fernandes, Juliana Campos Hasse Fernandes, Júlio César Joly

**Affiliations:** ^1^Department of Periodontology, São Leopoldo Mandic Research Institute, São Leopoldo Mandic, Campinas, SP, Brazil; ^2^Periodontics, Missouri School of Dentistry and Oral Health, A.T. Still University, St. Louis, MO, U.S.A.; ^3^Private Researcher. St. Louis, MO, USA

**Keywords:** Morbidity, Palate, Surgical closure technique, Tissue grafting, Wound healing

## Abstract

**Background.:**

Periodontal and peri-implant soft tissue management in oral rehabilitation is often necessary to achieve more esthetic and stable clinical results. This involves harvesting connective tissue from the palate. There is no consensus about the technique that will cause less postoperative pain in the donor area. Thus, this prospective cohort study compared the postoperative morbidity of two surgical techniques from the palate donor site: the free gingival graft (FGG)/de-epithelialized technique and the linear technique/subepithelial technique.

**Methods.:**

Sixteen patients were randomly assigned to the FGG/de-epithelialized removal group (G1) and the removal of the connective tissue graft (CTG) with the linear/subepithelial technique group (G2). The morbidity analysis consisted of measuring the number of anti-inflammatory agents taken in the postoperative period, pain analysis through a visual analog scale, and visual analysis of healing of palatal soft tissues 1, 2, and 3 weeks after surgery.

**Results.:**

The results showed that the G1 patients took more anti-inflammatory drugs (mean=9.88) than the G2 (mean=3.63) and experienced more postoperative pain (mean=6.38) than G2 (mean=3) (*P*<0.05 for both parameters). In the visual analysis of healing, the results were better for G1 on days 7 and 21; however, on day 14, the results were better for G2, with no significant differences (*P*>0.05) between the groups at any of the experimental times.

**Conclusion.:**

Both techniques promoted effective healing of the palatal area; however, the removal by the linear graft technique caused less postoperative pain.

## Introduction

 Given the increased aesthetic demand in dental treatments, soft tissue grafts have become essential tools for tissue reconstruction. Despite the unquestionable benefits, the literature reports that graft removal can cause problems, including the risk of postoperative complications and pain.^1^ The selection of the donor area for soft tissue grafts must consider tissue availability, risks to the patient’s health, and postoperative morbidity, seeking a risk-benefit ratio favorable to the patient and the success of the treatment.^2^ Among the possible intraoral areas of choice, we can highlight the palate and the maxillary tuberosity area. In general terms, the grafts from the different sites differ in their dimensions, with the tuberosity grafts being more voluminous, those from the posterior region of the palate being thinner, and those from the anterior region of the palate being more extensive.^2^ Also, each site has a unique gene expression profile, impacting its biological behavior and outcomes.^3^ Therefore, the palate is the region most frequently used to remove connective tissue grafts (CTGs) and free gingival grafts (FGGs).^4^

 Although this procedure is associated with a specific morbidity for the patient, CTG is still considered the “gold standard” for most reconstructive procedures.^5,6^ Therefore, adding another surgical area increases the complexity of the procedure and patient pain. Thus, choosing a surgical technique to remove tissue must consider the reduction of morbidity, patient acceptance,^7^ obtaining the largest volume of tissue, and minimizing pain and the risk of postoperative complications as much as possible.^2^ Many techniques for obtaining CTG have already been described, including the trapdoor,^8^ FGG/de-epithelialized,^9,10^ double-blade,^11^ double-incision,^12^ and linear or single-incision techniques.^13,14^

 FGG/de-epithelialized removal is the easiest way to achieve this, and it allows for the retrieval of a large amount of high-quality connective tissue. On the other hand, it produces a surgical site with the secondary intention of healing,^15^ which takes 2‒4 weeks to heal and is constantly associated with greater pain for the patient.^16^ It consists of making four incisions to remove the epithelial-connective tissue set. In this technique, the flap is not repositioned, leaving the wound to heal by secondary intention.^9,10^ Firstly, it was developed to be removed with the periosteum; more recently, it was recommended to be removed without involving the periosteum when the graft must be de-epithelialized for use only of the connective tissue.^4^ In a study conducted by Zucchelli et al.^17^ regarding the FGG technique to harvest CTG for root coverage, the findings showed that reducing the size of the CTG provided less morbidity for the patient without compromising clinical results.

 Another technique is the linear incision/subepithelial (sCTG).^9^ According to Hürzeler and Weng,^13^ the linear graft removal technique consists of making a single incision perpendicular to the long axis of the teeth, extending deep into the palate until the desired height is obtained. The second step involves a more superficial incision at the same point, made until it reaches the point where the first incision ended. This incision removes connective tissue with an approximate thickness of 1.5 mm, which must be carefully trimmed to remove adipose tissue and the beveled edges of the graft. This technique helps with healing and reduces postoperative morbidity in the donor area. It allows for primary closure and accelerates wound healing, reducing postoperative complications and improving the patient’s postoperative comfort.^14^ However, an adequate thickness of the palatal fibromucosa is required. It is a method of increasing the alveolar ridge in edentulous regions, described as a viable procedure for different root coverage techniques.^1^ Lorenzana and Allen^14^ also described a modified CTG removal technique for tissue reconstructions in which a single incision is made in the palate; thus, it allows a first-intention healing, causing less pain at the donor site during the recovery period.

 Therefore, the literature still lacks consensus regarding the most favorable technique for soft tissue grafts, specifically whether linear or de-epithelialized/FGG. Thus, this study clinically evaluated the morbidity of both graft removal techniques from the palatal region (FGG/de-epithelialized and linear/subepithelial technique), assessing the level of postoperative pain, anti-inflammatory consumption, and tissue healing. The null hypothesis was that the postoperative pain was similar when harvesting the CTG through both techniques.

## Methods

 The research was conducted after obtaining approval from the Research Ethics Committee (IRB) of the São Leopoldo Mandic - Faculty of Dentistry and the Center of Dental Research (protocol number 1.468.698). This prospective cohort study followed the Helsinki Declaration (1975, updated 2013) and STROBE guidelines; all the participants were assessed and recruited between 2021 and 2022 at the clinic of the São Leopoldo Mandic (Campinas, Brazil). They understood the study and signed the informed consent form before inclusion. All surgeries were performed by the same dental surgeon (J.C.J.), a specialist in periodontics.

###  Eligibility Criteria

 The following inclusion criteria were considered: (1) patients with the need for simple soft tissue reconstruction (up to two adjacent teeth); (2) acceptance to have the autogenous soft tissue–CTG, harvested from the palate region; (3) presence of palatal tissue availability based on clinical assessment.

 Smoking patients taking anti-inflammatory agents and/or antibiotics, with diabetes (any level) or other systemic condition, with plaque index > 20%, who had any contraindication to oral surgical procedure, pregnant or breast-feeding, and bleeding on probing (BoP) > 10% were excluded.

###  Sample Size

 A sample size of 8 patients per group was necessary to detect a minimum clinically significant difference of 3.4 intraperiod of FGG with 1.9 intraperiod of sCTG, for the pain assessment, using a two-tailed test of variance, α = 0.05, power of 80%, and standard deviation of 0.8.^1^ Observing the risk of dropout, we considered increasing the number of samples by 20% per group, totaling 10 patients/group.

###  Surgical Procedures

####  Free Gingival Graft (FGG)/De-Epithelialized Technique

**Figure 1:**
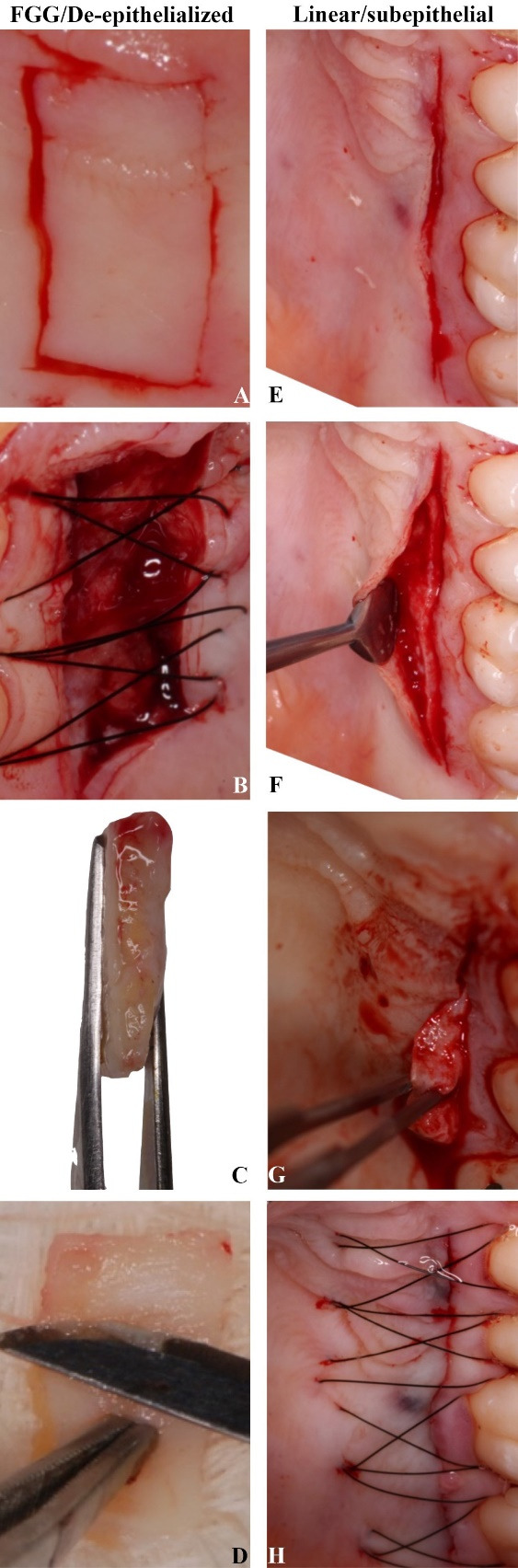
**(A)** Delimitation of the area of the graft to be removed with incisions perpendicular to the bone crest of the palate. **(B)** Donor area sutured. **(C)** Free gingival graft (FGG) removed showing the epithelium (left) and connective (right). **(D)** Removal of the epithelium to obtain and use only the connective tissue de-epithelialized. **(E)** Initial incision perpendicular to the palatal bone crest 2‒3 mm from the gingival margin. **(F)** Flap after division with a 15c scalpel blade (Swann-Morton) into the epithelial portion and underlying connective tissue. **(G)** Removal of the connective tissue with the presence of periosteum. **(H)** Compressive suture in 5-0 nylon suture (Ethicon)

 This technique of removing the CTG followed the description provided by Zucchelli et al.^4^ The first step involved assessing the dimensions required for tissue reconstruction, as well as the availability of the donor area. It was followed by the administration of local anesthesia (4% articaine with 1:100,000 epinephrine, DFL, Brazil). Then, two horizontal incisions were made (the most coronal was 2‒3 mm from the gingival margin) and two vertical incisions to delimit the area to be removed (Figure 1A). The blade was inserted perpendicular to the bone surface in the horizontal incision. Once enough tissue was reached, the blade was rotated to a parallel position to the tissue surface. Tissue thickness was maintained uniformly (around 1.5 mm) as the blade moved apically without removing the underlying periosteum. No protective material was placed on the bed, and a compressive suture was performed with 5-0 nylon thread (Ethicon) to maintain the fibrin layer and local hemostasis (Figure 1B). In this surgical approach, it is possible to observe the epithelial and connective tissue that has been removed (Figure 1C). Then, the epithelial portion was removed on the bench outside the mouth, supported by sterile gauze richly soaked in saline solution (Figure 1D).

####  Linear/Subepithelial Technique

 The linear incision removal/subepithelial technique followed the description of Lorenzana and Allen.^14^ The first step involved assessing the dimensions required for tissue reconstruction, as well as the availability of the donor area. Following the administration of local anesthesia (4% articaine with 1:100,000 epinephrine, DFL, Brazil), a blade oriented perpendicular to the surface of the palatal tissue made the initial incision. A single incision was made horizontally to the bone approximately 2‒3 mm apical to the gingival margin of the teeth, with the length of the incision being determined by the graft dimensions required. A partial dissection of the flap was performed within the single incision, leaving an adequate thickness of tissue to prevent sloughing of the overlying tissue. The dissection goes apically to the dimensions necessary to obtain the graft. The connective tissue with the periosteum was then carefully lifted with the help of a small elevator. Careful manipulation of the graft was done using delicate forceps. The flap was then closed with compressive suture in 5-0 nylon thread (Ethicon), which was removed 7 days after the procedure (Figure 1E-H).

###  Postoperative Care and Parameters Assessed

 Postoperative instructions included prescribing a 0.12% chlorhexidine digluconate solution as a mouthwash, to be used for one minute, twice daily, for 15 days. A liquid and/or soft diet with cold or iced foods was requested to be kept for 48 hours following surgery. Ibuprofen (600 mg) was prescribed only in case of pain, and the patients were asked to write the number of tablets ingested in the postoperative period according to the methodology used in previous studies by Wessel & Tatakis^1^ and Zucchelli et al.^4^

 The suture was removed 7 days after the procedure. Then, the patients were instructed to attend follow-up appointments 7 (A), 14 (B), and 21 days (C) postoperatively. During the first follow-up (7 days), the number of tablets taken that week was cataloged, and the suture was removed from the donor site (palate). During this same consultation, a questionnaire was administered to assess the patient’s pain using the visual analog scale (VAS), with values ranging from 0 (no pain) to 10 (extremely painful). The questionnaire was administered to measure postoperative pain during the week after surgery. The patients were asked to indicate the location of the pain (donor site, recipient site, or other areas).

 Regarding the analysis of the evolution of tissue healing in the donor area, the patients had their areas photographed with a digital camera (Canon t5i with Youngnuo circular flash) by the same operator (not involved in the evaluation) 7, 14, and 21 days postoperatively, with the early wound-healing index (EHI), first described by Wachtel et al^18^:

1. Completely closed flap, without fibrin line on the palate
2. Closed flap with fibrin line on the palate
3. Closed flap with small fibrin clots in the palate
4. Flap with incomplete closure with partial necrosis of the palate ( < 50% of the flap involved)
5. Flap with incomplete closure with total necrosis of the palate ( > 50% of the flap involved)

Two experienced professors performed all the evaluations (E.M.L. and G.V.O.F.) and the photographs individually; they were previously calibrated by analyzing the photographs from an article with a similar methodology^7^ that illustrates each index to be considered (k = 0.90). A third referee was consulted in case of any disagreement (J.C.H.F.). The images were sent to the evaluators to assign the indices. At the time of the evaluation, they were blinded to the groups and unaware of each other’s analysis.

###  Statistical Analysis

 The results were expressed through descriptive statistical measures, including mean, standard deviation, median, minimum, and maximum values. They were analyzed inferentially using the Mann-Whitney statistical test for comparing groups and the Friedman test for comparing assessment times. In cases of significant differences between evaluations, multiple comparison tests were used. To evaluate the degree of agreement between the evaluators regarding the visualization of healing, the observed agreement value, the weighted kappa value, and the confidence interval for that parameter were obtained. The margin of error used in the statistical test was 5.0%, and the interval was obtained with 95% confidence. The data were entered into an Excel spreadsheet, and statistical calculations were performed using SPSS software (Statistical Package for the Social Sciences, version 23).

## Results

**Figure 2:**
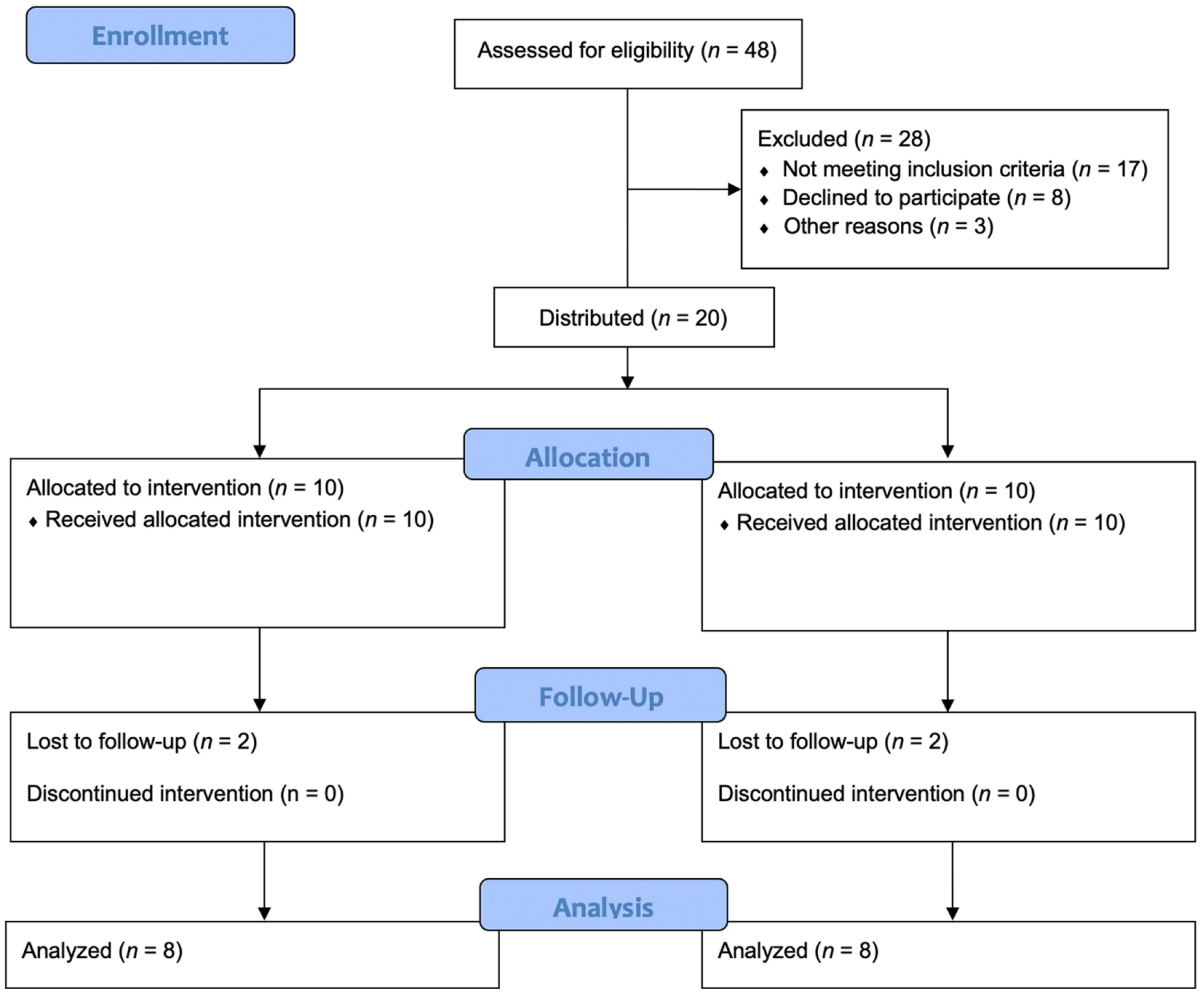
Diagram flow for the selection and inclusion of patients

 Forty-eight patients were initially evaluated. Therefore, 20 patients (mean age, 37 ± 7.8; 11 males and 9 females) were enrolled and divided into two groups based on their treatment: G1, free gingival graft/de-epithelialized (n = 10), and G2, linear/subepithelial technique (n = 10). Of the 20 patients operated on, there was a dropout/exclusion of four patients (3 males and 1 female) due to missing follow-up appointments (2 of them moved to another city, and 2 lost follow-up due to scheduling difficulties), which resulted in two groups, each comprising 8 patients (G1 [n = 8] and G2 [n = 8]) (Figure 2). No complications or adverse events were observed during the surgical procedures and follow-ups.

###  Visual Pain Scale and Number of Tablets Used

**Figure 3:**
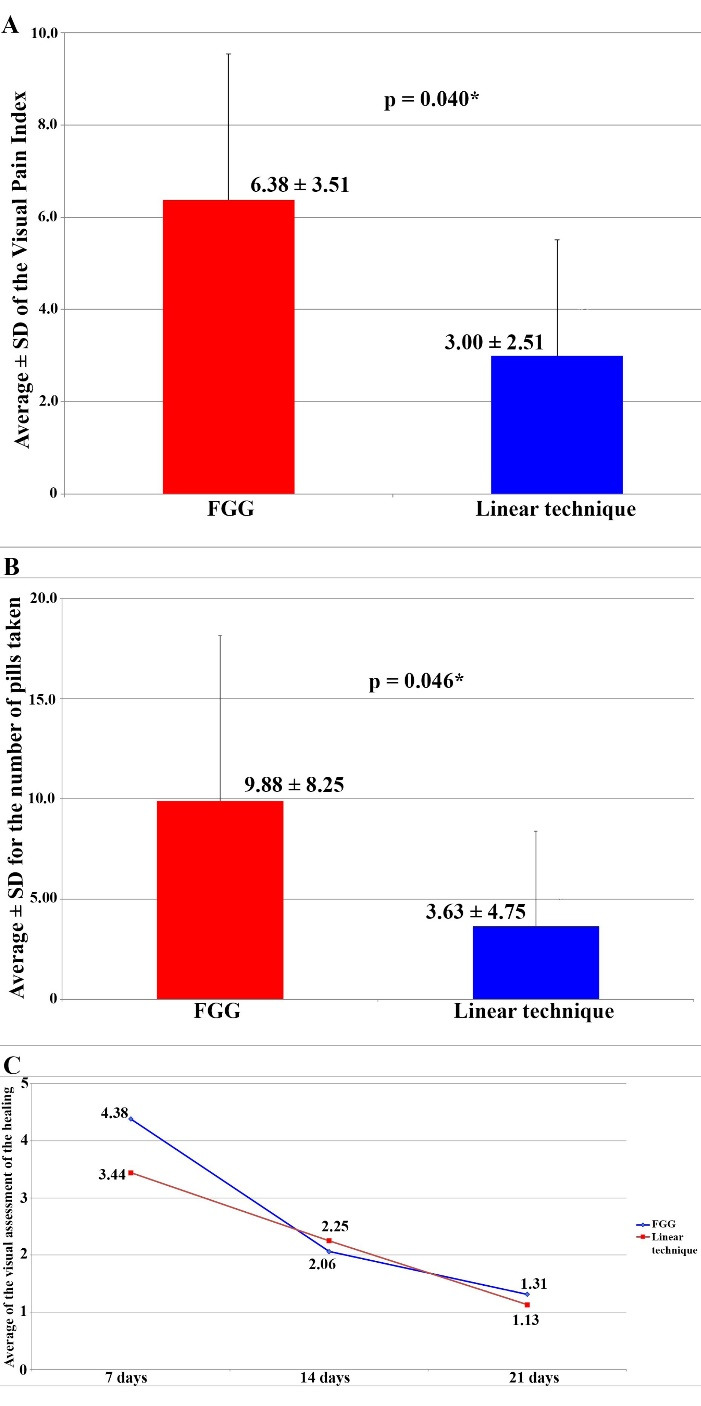
**(A)** Average ± SD for the visual pain index. **(B)** Average ± SD for the number of tablets taken. **(C)** Means of visual assessment of healing according to the group studied

Table 1 and Figure 3 present the results regarding the visual pain scale and the number of tablets used. The mean and median values of the visual pain scale were higher in G1 (FGG technique), with a mean of 6.38 ± 3.16 and a median of 7.0. The linear technique group (G2) exhibited an average of 3.0 ± 2.51 and a median of 2.50 (*P* = 0.040). The mean and median numbers of tablets used were also higher in G1, with a mean of 9.88 ± 8.25 and a median of 8.0, whereas in G2, the average was 3.63 ± 4.75, with a median of 2.0 (*P* = 0.046).

###  Visual Assessment of Healing

**Figure 4:**
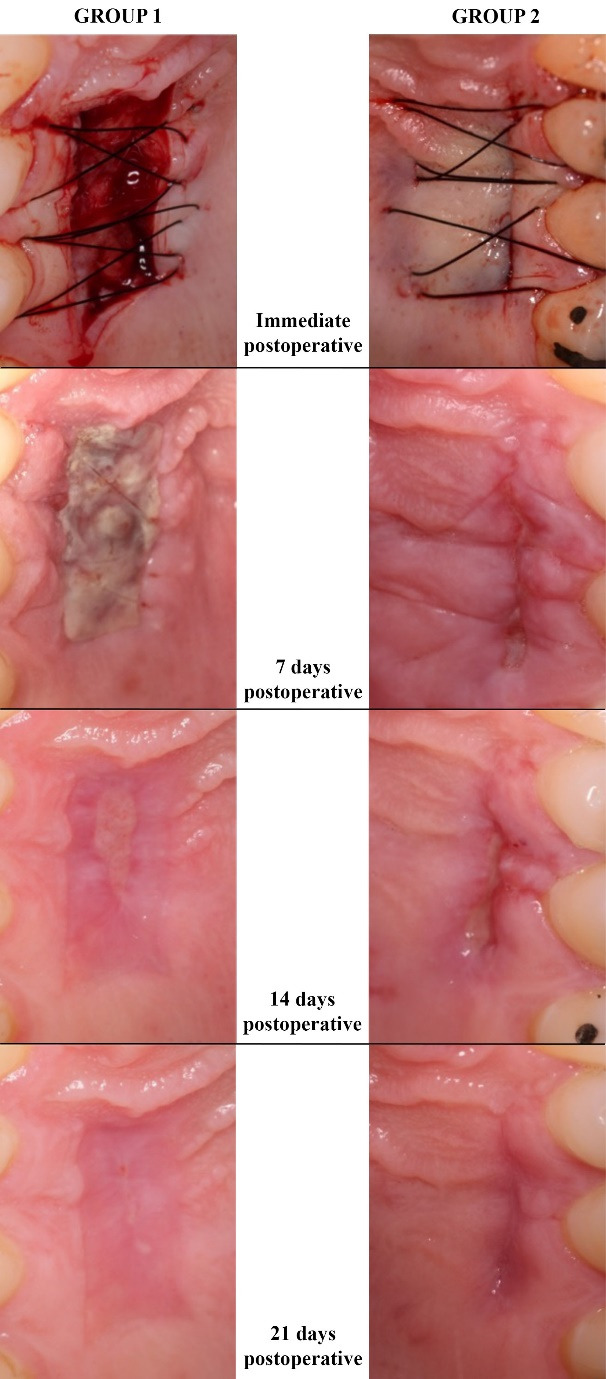
Visual assessment of the palatal healing according to the group studied. FGG/de-epithelialized technique (group 1) (left side) and linear/subepithelial technique (right side)

Figure 4 shows the immediate postoperative clinical appearance of both groups at 7, 14, and 21 days. Table 1 and Figure 3C present the results of the visual assessment of healing, considering the average of the two independent evaluators across the groups and follow-up periods. Between the groups, the means were higher in G1 (FGG) after 7 and 21 days, while after 14 days, the mean was higher in G2; however, no significant differences were observed (*P* > 0.05). In the 7-day evaluation, the means were 4.38 ± 0.88 and 3.44 ± 1.05 for G1 and G2, respectively (*P* = 0.067). After 14 days, the averages were 2.06 ± 0.94 in G1 and 2.25 ± 0.46 in G2 (*P* = 0.398). In the 21-day evaluation, G1 averaged 1.31 ± 0.53, with 1.13 ± 0.23 in G2 (*P* = 0.713). Regarding assessment periods, the means and medians obtained showed a reduction. In G1, the mean reduced from 4.38 to 1.31 and the median from 4.75 to 1.00, while in G2, the mean reduced from 3.44 to 1.13 and the median from 3.75 to 1.0 (*P* < 0.001).

 The agreement observed between the two evaluators, regardless of the group and evaluation time, was 30 (62.5%) in a total of 48 measurements. The weighted kappa value was 0.742 (good agreement), with a range of 0.633 to 0.851. Table 2 summarizes all the data.

**Table 1:** Statistical results for the visual pain index and number of tablets taken (top) and visual assessment of healing according to group and follow-up (bottom)

**Variable analyzed**	**Statistical result**	**FGG (n=8)**	**Linear technique (n=8)**	***P*** ** value**
Visual pain index	Average	6.38	3.00	*P* (1) = 0.040*
	SD	3.16	2.51	
	Median	7.00	2.50	
	Minimum	0	0	
	Maximum	10	8	
Number of tablets taken	Average	9.88	3.63	*P* (1) = 0.046*
	SD	8.25	4.75	
	Median	8.00	2.00	
	Minimum	1	0	
	Maximum	26	15	
**Follow-up**	**Statistical result**	**FGG (n=8)**	**Linear technique (n=8)**	***P*** ** value**
7 days	Average	4.38 (A)	3.44 (A)	*P* (1) = 0.067
	SD	0.88	1.05	
	Median	4.75	3.75	
	Minimum	3.00	2.00	
	Maximum	5.00	5.00	
14 days	Average	2.06 (B)	2.25 (B)	*P* (1) = 0.398
	SD	0.94	0.46	
	Median	1.75	2.25	
	Minimum	1.00	1.50	
	Maximum	4.00	3.00	
21 days	Average	1.31 (C)	1.13 (C)	*P* (1) = 0.713
	SD	0.53	0.23	
	Median	1.00	1.00	
	Minimum	1.00	1.00	
	Maximum	2.50	1.50	
*P* value		*P* (2) < 0.001*	*P *(2) < 0.001*	

(1) = used Mann-Whitney test; (2) = used Friedman’s test; SD = Standard deviation; A,B,C = subgroups (period); SD = Standard deviation; * = significant difference (*P* < 0.05); (1) = using the Mann-Whitney test.

**Table 2:** Complete parameter data per patient, visual pain index, number of tablets used, and visual assessment of healing (averages of the two examiners)*

**Patient**	**Pain index (visual analogue scale)**	**Medication**	**Visual analysis of healing**					
			**Appraiser 1**			**Appraiser 2**		
			**7 days**	**14 days**	**21 days**	**7 days**	**14 days**	**21 days**
G1.1	6	10	5	2	2	5	2	1
G1.2	10	11	5	2	1	5	1	1
G1.3	8	3	3	2	1	3	1	1
G1.4	5	17	3	1	1	3	1	1
G1.5	0	26	5	3	1	4	2	1
G1.6	5	6	5	5	3	4	3	2
G1.7	8	5	5	3	2	5	2	1
G1.8	9	1	5	2	1	5	1	1
G2.1	1	0	3	2	1	3	2	1
G2.2	3	4	4	2	1	4	2	1
G2.3	8	3	5	3	1	5	2	1
G2.4	0	2	4	3	2	4	2	1
G2.5	2	2	4	3	2	3	2	1
G2.6	2	15	2	2	1	2	1	1
G2.7	5	2	2	2	1	2	2	1
G2.8	3	1	4	3	1	4	3	1

*Group 1 (blue) - free gingival graft/de-epithelialized technique and group 2 (red) - Linear/subepithelial technique. Pain index (visual analogue scale): ranged from 0 to 10; Visual analysis of healing: ranged from 1 to 5.

## Discussion

###  Surgical Techniques and Complications

 The present study observed the patient’s postoperative pain after harvesting the CTG through the FGG/de-epithelialized or linear incision/subepithelial techniques. FGG presented a higher pain level than the linear technique, with a statistically significant result, consistent with a previous study.^1^ These authors compared the removal of the CTG between the same techniques; they treated 23 patients, 12 with subepithelial CTG and 11 with FGG/de-epithelialized, and on the third postoperative day, the proportion of patients who reported pain in the palate was significantly higher for the FGG group. Griffin et al^19^ found similar results, showing that patients undergoing FGG procedures had a greater probability of bleeding and edema than those undergoing sCTG (linear technique). Del Pizzo et al^20^ also evaluated tissue repair after harvesting from the palate using FGG and sCTG; they found a significantly lower postoperative morbidity result when the graft was removed using the sCTG technique. In contrast, Zucchelli et al^4^ conducted a comparative study between collecting FGG and the trapdoor technique, which has two releasing incisions.^8^ They comparatively evaluated the morbidity between the two procedures and found no statistically significant difference between them.

 Moreover, the linear incision technique was developed and described as having more favorable postoperative morbidity control than the trap door, as it does not have releasing incisions, favoring the blood supply for tissue healing.^14^ If such results were found when comparing these two techniques, we can infer that with an even less invasive technique (linear incision), these results would be even more discrepant, favoring the linear incision.

 Among the various documented techniques for harvesting connective tissue from the palate, it is essential to remember that when choosing one, the professional should prefer the method that causes the least pain, as it is the second surgical site to be addressed. The present study revealed a difference in postoperative pain, which favors removal using the linear incision/subepithelial technique. In our opinion, the choice of technique depends on factors such as the patient’s behavioral profile, surgical time, skill, and level of experience of the operator, as well as tissue availability in the donor area. In patients with adequate soft tissue thickness, it is recommended that tissue removal be performed using a less invasive technique, which promotes better postoperative comfort. In patients with limited tissue availability or operated by less trained professionals, the FGG/de-epithelialized technique can be a good choice due to its greater ease of execution and the increased risk of only a thin layer of epithelium remaining covering the wound if the other technique is applied, which can cause necrosis of the local tissue.

 It is essential to highlight the importance of adequate keratinized tissue width (KTW) around dental implants and teeth soft tissue and volume, particularly in the vertical and buccolingual dimensions, which are essential for achieving a favorable emergence profile, contributing to the esthetic appeal of the restoration, and better local protection against bacteria,^21^ due to the increased resistance.^22^ The importance of these variables, such as KTW, was indirectly correlated to the marginal bone loss (MBL) and probing depth (PD); in the case of an adequate volume of KTW, lower PD and MBL were found.^23^ Moreover, some evolutions and advances were found for periodontal surgeries, which occurred with the implementation of microscopes/augmentation loupes. Khan et al^24^ showed that microsurgery results in faster healing and a predictable outcome, suggesting reduced trauma, which may allow a quicker suture removal without jeopardizing the outcomes. Therefore, when assessing the efficacy of macro- and micro-surgical procedures in removing the epithelial tissue layer of the CTGs, the authors concluded that samples harvested by micro-surgery had greater remaining epithelial portions observed than those harvested by macro-surgery (*P* = 0.57), with similar connective layer thickness.^25^ This fact was corroborated by Maia et al,^26^ who concluded there was incomplete removal of the epithelial layer after harvesting the CTG of 44.32% due to its histological persistence, suggesting the clinical removal was inaccurate, independently of the professional experience.

 In addition, although no adverse event was observed, it is essential to highlight the average to achieve the greater palatine artery of 12 mm (a range of 9 to 16 mm) that, in most cases, was found at a distance of 76% of the height of the palate, measuring from the cementoenamel junction of the first molar.^27^ Also, complications of the healing process after FGG and sCTG removal procedures were reported in several studies.^28-30^ The most reported complications resulting from the removal of the FGG are hemorrhage, herpetic lesions, paresthesia, mucocele, bone exposure, and postoperative pain.^28,31^ Complications resulting from subepithelial connective tissue grafting (sCTG) include excessive bleeding, graft retraction, necrosis of the graft and palatal tissue, pain, and infection in the donor and/or recipient area,^32^ with necrosis of the donor area being the primary concern, due to the lack of adequate thickness of the fibromucosa and failure in primary closure.^8,33^

###  Pain Evaluation

 The average pain VAS in the present study was 3 for the linear technique, which is close to the value reported by other authors,^1^ who showed 3.5 on the third day after the surgical procedure. For the FGG group, the average pain VAS found in this study was 6.38, while the authors reported 4.8 on the third postoperative day. Moreover, Marques et al^34,35^ performed a 3D digital analysis of the hard palate wound healing after FGG, concluding that the palatal wound region’s mean thickness reduced by −0.26 ± 0.31 mm after three months.

 In the present study, postoperative pain findings indicated that the pain reported by patients operated on using the FGG technique was 2.13 times greater than that using the linear incision technique (6.38 vs. 3.00). Griffin et al^19^ compared FGG removal and subepithelial CTG removal using the two parallel incision techniques.^12^ The results showed that patients who underwent FGG were 3 times more likely to develop postoperative pain (*P* = 0.002) or bleeding (*P* = 0.03) compared to those who received the linear technique of removal, concluding that the FGG group had a greater risk of postoperative pain or bleeding. Although we did not specifically evaluate the risk of postoperative bleeding, the pain findings were similar. Moreover, the greater risk of pain and bleeding may be associated with the presence of only one compressive suture to maintain the clot in the FGG technique. In contrast, in the other technique, the epithelium layer protects the surgical bed.

 Zucchelli et al^4^ compared two forms of graft removal, FGG and the trapdoor (TD) technique. They did not find a significant difference in relation to the use of analgesics in the postoperative period, nor was a significant difference found in the pain VAS analysis. The patients were also assessed for difficulty chewing and postoperative stress, and in these two evaluations, the results were statistically significant, with favorable outcomes for the trapdoor technique. The authors found a significant difference regarding analgesic use when there was necrosis of the trapdoor flap, compared to when the FGG was removed. Therefore, the authors considered the importance of evaluating the thickness of the palate when selecting the appropriate technique. It is recommended that if a chosen technique attempts healing by first intention, the site should have sufficient tissue thickness. After removing the CTG, there should still be enough connective tissue to avoid dehiscence/necrosis of the flap.

###  Healing Process

 The present study compared tissue healing in the donor area of soft tissue grafts using an early tissue healing index (EHI) first described by Wachtel et al^18^ and modified by Fickl et al.^7^ The findings here show better healing for the linear incision technique on days 7 and 21, while on day 14, there was better healing for the FGG technique (not statistically significant). Regarding healing during the evaluation period, a statistically significant difference was observed between the two groups (*P* < 0.001). These findings suggest that, regardless of the technique chosen, the palate undergoes good regeneration over time, making this area an acceptable donor site. Del Pizzo et al^20^ evaluated palate healing by comparing the FGG, TD, and single/linear incision (SI) techniques in 36 patients; in all groups, 100% of patients had total epithelialization of the area within 4 weeks.

 When comparing the results of the healing VAS and the quantity of pills ingested by patients, a variation was noted between the number of tablets taken and the pain reported by the patient. However, the data from this study suggest a correlation between the number of tablets ingested and the pain experienced by the patient. In G1, patients who reported less pain ingested more 600-mg Ibuprofen tablets, while patients who reported more pain took fewer tablets. This relationship was also found in G2, but the values for the number of tablets and reported pain were significantly lower.

## Limitations

 The present cohort study included a limited number of patients. The results could be different, and a statistically significant difference would likely be found in the 7-day evaluation period if the study had included a larger sample of patients. Unfortunately, as with every clinical study, this one had its limitations; some patients failed to attend the follow-up appointments, which reduced the sample size and limited the significance of the results. During the 14- and 21-day healing periods, a significant difference would probably not be found, even with a larger sample, due to the proximity of the values found in the results of this study. Additionally, patients’ reports of pain have a certain degree of subjectivity, as each individual has a unique pain threshold and responds to painful stimuli in their own way; moreover, some patients feel more comfortable taking medications to alleviate pain, while others prefer to endure the painful sensation rather than use medications.

## Conclusion

 Despite this study’s limitations, it was possible to conclude that graft removal using the linear/subepithelial technique caused significantly less postoperative pain and morbidity. Therefore, both methods effectively healed the palatal area with no differences. It was possible to reject the null hypothesis because removing the graft in a less invasive procedure and maintaining the local epithelial portion (in the palate) had a positive relationship with the degree of comfort felt by the patient in the postoperative period.

## Competing Interests

 Theauthors declare that they have no conflicts of interest related to this study.

## Data Availability

 All data is available in the study.

## Ethical Approval

 São Leopoldo Mandic - Faculty of Dentistry and Center of Dental Research approval (protocol number 1.468.698).
